# Association between incident delirium and 28- and 90-day mortality in critically ill adults: a secondary analysis

**DOI:** 10.1186/s13054-020-02879-6

**Published:** 2020-04-20

**Authors:** Matthew S. Duprey, Mark van den Boogaard, Johannes G. van der Hoeven, Peter Pickkers, Becky A. Briesacher, Jane S. Saczynski, John L. Griffith, John W. Devlin

**Affiliations:** 1 Department of Pharmacy and Health Systems Sciences, Bouve College of Health Sciences, Northeastern University, 360 Huntington Ave, Boston, MA 02115 USA; 2 Department of Intensive Care, Radboud Institute for Health Sciences, Radboud University Medical Center, P.O. Box 9101, 6500 HB Nijmegen, the Netherlands; 3 Department of Health Sciences, Bouve College of Health Sciences, Northeastern University, 360 Huntington Ave, Boston, MA 02115 USA

**Keywords:** Delirium, Mortality, Intensive care, Coma, Risk factors

## Abstract

**Background:**

While delirium prevalence and duration are each associated with increased 30-day, 6-month, and 1-year mortality, the association between incident ICU delirium and mortality remains unclear. We evaluated the association between both incident ICU delirium and days spent with delirium in the 28 days after ICU admission and mortality within 28 and 90 days.

**Methods:**

Secondary cohort analysis of a randomized, double-blind, placebo-controlled trial conducted among 1495 delirium-free, critically ill adults in 14 Dutch ICUs with an expected ICU stay ≥2 days where all delirium assessments were completed. In the 28 days after ICU admission, patients were evaluated for delirium and coma 3x daily; each day was coded as a delirium day [≥1 positive Confusion Assessment Method for the ICU (CAM-ICU)], a coma day [no delirium and ≥ 1 Richmond Agitation Sedation Scale (RASS) score ≤ − 4], or neither. Four Cox-regression models were constructed for 28-day mortality and 90-day mortality; each accounted for potential confounders (i.e., age, APACHE-II score, sepsis, use of mechanical ventilation, ICU length of stay, and haloperidol dose) and: 1) delirium occurrence, 2) days spent with delirium, 3) days spent in coma, and 4) days spent with delirium and/or coma.

**Results:**

Among the 1495 patients, 28 day mortality was 17% and 90 day mortality was 21%. Neither incident delirium (28 day mortality hazard ratio [HR] = 1.02, 95%CI = 0.75–1.39; 90 day mortality HR = 1.05, 95%CI = 0.79–1.38) nor days spent with delirium (28 day mortality HR = 1.00, 95%CI = 0.95–1.05; 90 day mortality HR = 1.02, 95%CI = 0.98–1.07) were significantly associated with mortality. However, both days spent with coma (28 day mortality HR = 1.05, 95%CI = 1.02–1.08; 90 day mortality HR = 1.05, 95%CI = 1.02–1.08) and days spent with delirium or coma (28 day mortality HR = 1.03, 95%CI = 1.00–1.05; 90 day mortality HR = 1.03, 95%CI = 1.01–1.06) were significantly associated with mortality.

**Conclusions:**

This analysis suggests neither incident delirium nor days spent with delirium are associated with short-term mortality after ICU admission.

**Trial registration:**

ClinicalTrials.gov, Identifier NCT01785290 Registered 7 February 2013.

## Background

Delirium occurs in up to 50% of critically ill adults, is associated with substantial burden to patients and their families, and is associated with serious ICU and post-ICU complications [1, 2]. Mortality after critical illness remains an important concern among ICU survivors and their families [3, 4]. Cohort studies evaluating the association either between delirium occurrence or days with delirium and mortality (either during or after ICU discharge) have reported inconsistent results (Additional file 1) [5–20]. Inclusion of patients with prevalent delirium (i.e., delirium occurrence either before or during ICU admission) rather than just incident delirium (i.e., delirium occurrence after ICU admission) in these analyses, where the occurrence of each is not distinguished, may account for the observed heterogeneity of assocation between delirium and mortality [5, 6]. Moreover, the way in which risk factors for both delirium and mortality, such as severity of illness, often change both before and during the ICU stay raise questions about the delirium-mortality relationships reported. For example, when daily ICU severity of illness was incorporated in one cohort study of 1112 critically ill adults, the association between ICU delirium and ICU mortality evaporated [6].

Neurologic status (i.e., coma, awake without delirium, or awake with delirium) often fluctuates on a day-by-day basis in the ICU. Coma is associated with increased mortality and is an independent risk factor for delirium occurrence, particularly when induced by a sedating medication [7–10]. This relationship is particularly well established within the first 48 h of ICU admission [10–12]. The well-established interaction between coma, delirium, and mortality makes it vital to consider the presence of coma in all ICU delirium-mortality evaluations; most reports to date have not considered it [5, 10, 13–19]. Models that have controlled for coma as either a time-dependent covariate [7, 20, 21], a competing risk [6], or as a simple indicator variable [22] have yielded different estimates regarding the effect of coma on the association between delirium and mortality in critically ill adults.

The primary aim of the study was to determine the association between incident ICU delirium and short-term mortality. As a secondary objective, we also sought to evaluate the association between the combined number of delirium and coma days and mortality at both 28 days and 90 days.

## Methods

### Study design and population

**Fig. 1:**
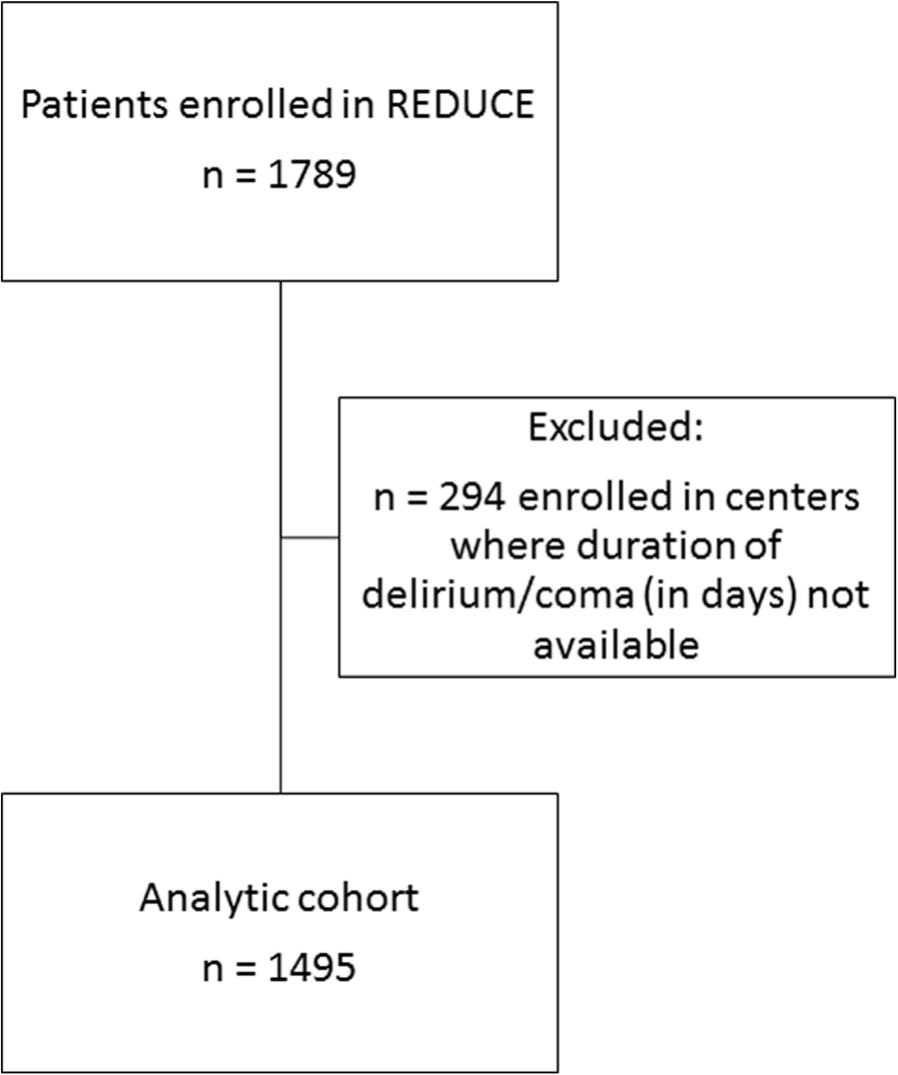
Flowchart for inclusion within the analytic cohort

This is a secondary cohort analysis of a three-arm randomized, placebo-controlled trial evaluating the efficacy of prophylactic haloperidol treatment in critically ill patients free from delirium at ICU admission. The study design and results have been previously described [23, 24]. In short, 1789 delirium-free, critically ill adults from 21 Dutch ICUs without acute neurologic injury who were expected to have an ICU length of stay ≥2 days were enrolled within 24 h of ICU admission and randomized to receive haloperidol 2 mg IV q8h, haloperidol 1 mg IV q8h, or placebo for up to 28 days or until delirium development, ICU discharge, or death (whichever occurred first). Among the 1789 patients enrolled in the parent study, 294 were cared for in centers where daily data regarding delirium and coma status was not available and thus excluded from this study (Fig. 1). For this analysis, all 3 arms of the trial were merged into a single cohort given the lack of reported difference in either 28- or 90-day mortality between the two haloperidol and placebo study arms. Data regarding duration of delirium and coma was only available for 14 of the 21 study centers. These 14 centers thus composed the analytic cohort of 1495 (83.6%) ICU patients [24]. This study was approved by the Arnhem-Nijmegen medical ethics committee (CMO-number 2012/424).

### Outcomes

Patients were assessed three times daily for the presence of coma (Richmond Agitation Sedation Scale [RASS] ≤ − 4 [25]) and delirium (if coma was not present) using the Confusion Assessment Method for the Intensive Care Unit (CAM-ICU) [26] for 28 days (or until death if it occurred prior to 28 days). Delirium was deemed to be present on any day if ≥1 delirium assessment was positive. A patient without coma or delirium on a particular day was deemed to be delirium- and coma-free on that day. Mortality was evaluated at both 28 and 90 days after enrollment. Baseline information including age, severity of illness [Acute Physiology and Chronic Health Evaluation II (APACHE-II) score] [27], presence of sepsis, requirement for mechanical ventilation, length of ICU stay, and receipt of haloperidol were also collected.

### Data analysis

Baseline characteristics were compared between the delirium and delirium-free groups. Continuous variables were compared using the Student’s t-test or Mann-Whitney U, according to distribution. Dichotomous variables were analyzed using the Chi-square test. A series of four Cox regression (either proportional hazards or linear, depending on predictor) models were created to model the hazard of death at 28 and 90 days after ICU admission. Four co-variables known to be associated with mortality in critically ill adults (i.e., age, APACHE-II score, presence of sepsis and use of mechanical ventilation) were identified a priori and subsequently forced into each model [27–29]. Additionally, receipt of haloperidol was controlled for in the analysis. Delirium and coma were modeled in 4 different ways: 1) delirium ever vs. delirium never; 2) number of days with delirium; 3) number of days with coma; and 4) number of days spent in delirium and/or coma. For each model, duration of ICU stay was a cumulative time-varying covariate. Models 1 and 2 did not control for coma status while model 3 did not control for delirium status. As a sensitivity analysis, length of ICU stay was added to each model as a competing risk factor. Additionally, a sensitivity analysis controlling for random patient allocation to haloperidol 2 mg IV TID, haloperidol 1 mg IV TID, or placebo was conducted to study the impact of randomization on the association in question. We evaluated interaction terms between trial arm and neurologic status (delirium or coma) within these models. A third sensitivity analysis was conducted to study the impact of coma occurring within the first 48 h. We also conducted additional analyses not controlling for mechanical ventilation and controlling for its duration. Model stability was tested by removing non-significant predictors after model completion; the fit of the model was evaluated using Akaike Information Criteria values [30]. All statistical tests were two sided and a *P* value < 0.05 defined statistical significance. All data was analyzed using SPSS version 24 (SPSS, Chicago, IL, USA).

## Results

**Fig. 2:**
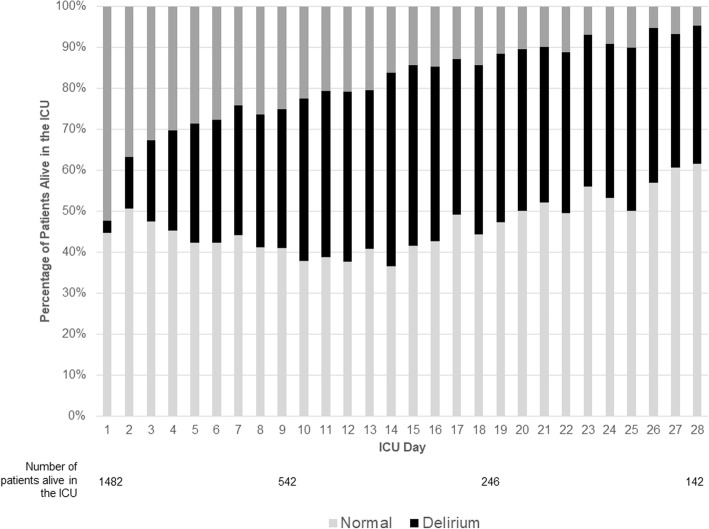
Graphical representation of the neurologicstatus for the remaining patients alive and in the ICU on each ICU day for the 28 days after ICU admission. Each patient was coded to one of three states [delirium, coma, or neither delirium nor coma (normal)]

Characteristics of the 1495 patients included in this analysis are presented in Table 1. Delirium developed during the ICU stay in 542 (36.2%) patients and 922 (61.7%) patients spent ≥1 ICU day with coma. In the 28 days after ICU admission, patients with delirium (*n* = 542) spent a median of 3 [first-third quartile (IQR) 1, 6] days with delirium, patients with coma (*n* = 922) spent a median of 2 [IQR 1, 5] days with coma, and patients who developed both (*n* = 369) spent a median of 7 [IQR 4, 12] days with coma or delirium. During the 28 days after ICU admission, the proportion of patients alive in the ICU with delirium, with coma, or with neither is presented in Fig. 2. A total of 185/1495 (12.4%) patients died in the ICU and 251/1495 (16.8%) and 308/1495 (20.6%) were dead by days 28 and 90, respectively.

**Table 1:** Patient charateristics

Variable	Total cohort (*N* = 1495)
Age in years, mean (SD)	66.3 (12.6)
APACHE II score, mean (SD)	19.2 (7.0)
Sepsis, N (%)	467 (31.2%)
Mechanical ventilation, N (%)	1156 (77.3%)
Delirium-positive, N (%)	542 (36.2%)
Days of delirium (in 28 days), median (IQR)	3 (1, 6)
Coma-positive, N (%)	922 (61.7%)
Coma days (in 28 days), median (IQR)	2 (1, 5)
Both delirium and coma, N (%)	369 (24.7%)
Delirium and coma days (in 28 days), median (IQR)	7 [4, 12]
Death in the ICU, N (%)	185 (12.4%)
Death within 28 days, N (%)	251 (16.8%)
Death within 90 days, N (%)	308 (20.6%)

Results are presented as n (%), mean ± SD, or median [IQR; first and third quartile]. Coma = RASS = − 4 or − 5. APACHE II the Acute Physiology and Chronic Health Evaluation II [25], RASS Richmond Agitation-Sedation Scale [23]

### Unadjusted models

**Fig. 3:**
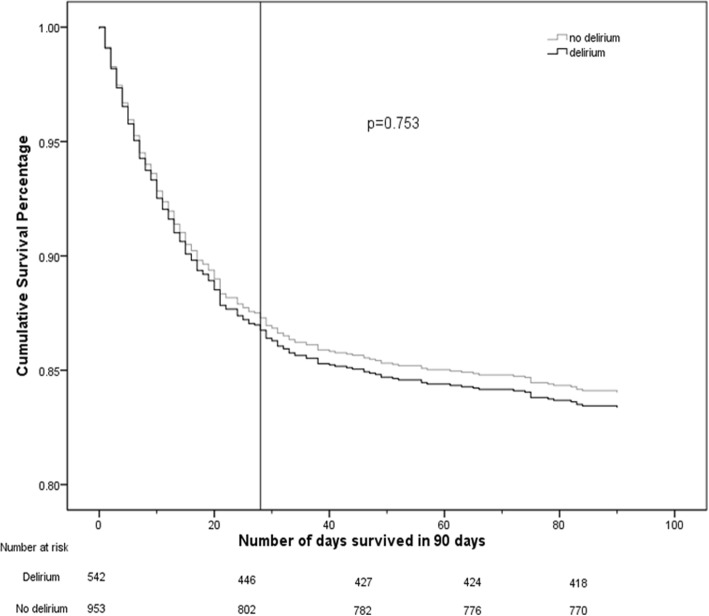
Survival curve over 28 and 90 days after ICU admission stratified by presence of incident delirium

**Fig. 4:**
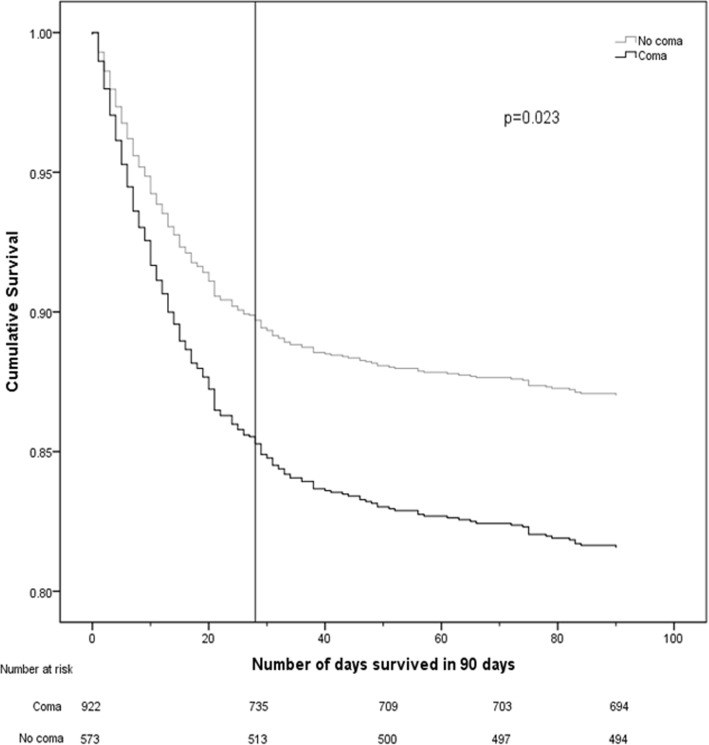
Survival curve over 28 and 90 days after ICU admission stratified by presence of coma

When potential confounders were not accounted for, neither incident delirium after ICU admission [28 day mortality, HR = 1.09; 95% Confidence Interval (CI) = 0.85–1.41; 90 day mortality, HR =1.18; 95% CI = 0.94–1.48) nor days with delirium in the 28 days after ICU admission (28 day mortality, HR = 0.99; 95% CI = 0.96–1.02; 90 day mortality, HR = 1.01; 95% CI = 0.98–1.04) were associated with short-term mortality (Fig. 3). Without adjusting for confounding, days with coma (in the 28 days after ICU admission) were associated with increased mortality at both 28- (HR = 1.06; 95% CI = 1.04–1.09) and 90- (HR = 1.07; 95% CI = 1.04–1.09) days (Fig. 4). The combination of days spent with delirium or coma (in 28 days) was associated with increased mortality at both 28 (HR = 1.02; 95% CI = 1.01–1.04) and 90 (HR = 1.03; 95% CI = 1.02–1.05) days.

### Models adjusted for confounding

After adjustment for potential confounding factors, incident delirium after ICU admission was not associated with mortality at either 28 (HR = 1.02; 95% CI = 0.75–1.39) or 90 (HR = 1.05, 95% CI = 0.79–1.38) days (Table 2). Total days spent with delirium in the 28 days after ICU admission was also not associated with mortality at either 28 (HR = 1.00, 95% CI = 0.95–1.05) or 90 (HR = 1.02, 95% CI = 0.98–1.07) days. Total days spent with coma in the 28 days after ICU admission was associated with mortality at 28 (HR = 1.05; 95% CI = 1.02–1.08) and 90 (HR = 1.05; 95% CI = 1.02–1.08) days. Days spent with either delirium or coma in the 28 days after randomization was also associated with mortality at 28 (HR = 1.03; 95% CI = 1.003–1.05) and 90 (HR = 1.03; 95% CI = 1.01–1.06) days. The results of these analyses are robust to the inclusion of length of ICU stay as a competing risk factor. Each of the mortality covariates (with the exception of ICU length of stay) was associated with increased 28- and 90-day mortality (Table 3).

**Table 2:** Estimation of the association between the four unique indicators of mental status over the ICU admission and both 28- and 90-day mortality

Variable	28-day mortality			90-day mortality		
	Unadjusted effect	Adjusted model 1*	Adjusted model 2^	Unadjusted effect	Adjusted model 1*	Adjusted model 2^
Incident Delirium	1.09 (0.85–1.41)	1.02 (0.75–1.39)	0.91 (0.68–1.21)	1.18 (0.94–1.48)	1.05 (0.79–1.38)	0.92 (0.71–1.19)
Days of Delirium	0.99 (0.96–1.02)	1.00 (0.95–1.05)	0.98 (0.94–1.02)	1.01 (0.98–1.04)	1.02 (0.98–1.07)	0.99 (0.96–1.03)
Days of Coma	1.06 (1.04–1.09)	1.05 (1.02–1.08)	1.19 (1.14–1.24)	1.07 (1.04–1.09)	1.05 (1.02–1.08)	1.12 (1.08–1.16)
Days of Delirium or Coma	1.02 (1.01–1.04)	1.03 (1.003–1.05)	1.07 (1.04–1.11)	1.03 (1.02–1.05)	1.03 (1.01–1.06)	1.05 (1.02–1.08)

Data is present as hazard ratios with their associated 95% confidence intervals. *Model 1 is adjusted to control for patient age, APACHE-II score at baseline, presence of sepsis, mechanical ventilation use, and haloperidol dose received, ^Model 2 is adjusted to control for patient age, APACHE-II score at baseline, presence of sepsis, mechanical ventilation use, haloperidol dose received, and ICU length of stay for each patient

**Table 3:** Estimates of the effect of covariates on 28- and 90-day mortality as evaluated in the eight Cox proportional hazards models

Variable	Mortality at 28 days				Mortality at 90 days			
Model	Incident delirium	Days of delirium	Days of coma	Days of delirium or coma	Incident delirium	Days of delirium	Days of coma	Days of delirium or coma
Age	1.04 (1.03–1.06)	1.04 (1.03–1.06)	1.05 (1.03–1.06)	1.04 (1.03–1.05)	1.04 (1.03–1.05)	1.04 (1.03–1.05)	1.04 (1.03–1.05)	1.04 (1.03–1.05)
APACHE II score	1.07 (1.06–1.09)	1.07 (1.06–1.09)	1.07 (1.05–1.09)	1.07 (1.06–1.09)	1.07 (1.05–1.08)	1.07 (1.05–1.08)	1.06 (1.05–1.08)	1.06 (1.05–1.08)
Sepsis present	1.89 (1.46–2.43)	1.87 (1.45–2.42)	1.73 (1.33–2.24)	1.87 (1.45–2.42)	1.86 (1.48–2.33)	1.85 (1.47–2.33)	1.72 (1.36–2.17)	1.78 (1.41–2.25)
Mechanical ventilation use	2.81 (1.88–4.19)	2.77 (1.86–4.13)	2.24 (1.50–3.36)	2.49 (1.66–3.72)	2.16 (1.54–3.05)	2.14 (1.53–3.00)	1.83 (1.30–2.58)	1.95 (1.38–2.74)
ICU Length of Stay	0.97 (0.95–0.99)	0.97 (0.96–0.99)	0.92 (0.90–0.95)	0.95 (0.92–0.97)	1.00 (0.98–1.01)	1.00 (0.98–1.01)	0.97 (0.95–0.99)	0.98 (0.97–1.00)
Study Arm								
Haloperidol 2 mg	1.08 (0.78–1.49)	1.06 (0.76–1.47)	1.20 (0.87–1.66)	1.29 (0.93–1.78)	1.17 (0.88–1.56)	1.18 (0.89–1.58)	1.31 (0.98–1.74)	0.97 (0.76–1.25)
Haloperidol 1 mg	1.18 (0.84–1.66)	1.16 (0.83–1.64)	1.12 (0.79–1.58)	1.21 (0.86–1.71)	1.15 (0.84–1.57)	1.15 (0.84–1.57)	1.16 (0.85–1.60)	0.99 (0.73–1.35)

Data is present as hazard ratios with their associated 95% confidence intervals

### Sensitivity analysis

Inclusion of ICU length of stay within the analytic models did not affect the nature of the association between delirium, coma, or both and mortality within our study (Table 2). Additionally, inclusion of prophylactic haloperidol treatment allocation within the model had a negligible effect on the effect size estimates presented (Table 3). There was no interaction between trial arm and neurologic status (Additional file 2). Coma occurring within the first 48 h of admission was not associated with changes in 28-day (HR = 1.12; 95% CI = 0.83–1.51) or 90-day (HR = 1.15; 95% CI = 0.87–1.53) mortality (Additional file 3). Models not controlling for length of stay or mechanical ventilation showed no change in effect size or significance (Additional file 4). Controlling for duration of mechanical ventilation, instead of just its presence, also failed to alter the model outputs (Additional file 5).

## Discussion

This large-scale investigation represents the first published study evaluating the effect of incident delirium on short-term mortality in critically ill adults admitted to the ICU. Our analyses indicate that incident ICU delirium, as well as the days spent with it, may not affect the likelihood of dying in the first 3 months after ICU admission. However, it does not preclude the importance of carefully evaluating all patients who develop delirium after ICU admission for the presence of possible risk factors for it, addressing those that are modifiable, and applying evidence-based delirium reduction strategies [31, 32]. Features of delirium such as fear and hallucinations may be distressing to patients [33] and the association between delirium and other long-term outcomes like post-intensive care syndrome are still being evaluated [32]. Nevertheless, questions still exist regarding whether mortality is the most appropriate, patient-centered endpoint for interventional studies focused on reducing delirium.

Our analyses also demonstrate that an association between coma and mortality exists in critically ill adults. This may be an epiphenomenon, and the associations we report do not imply a cause-effect relationship. Nevertheless, sedation-awakening trials have shown that deeper levels of sedation are associated with longer times to extubation and increased rates of tracheostomy, despite not having a proven association with 90-day mortality [31]. It is important to note that certain disease states excluded from our analysis such as traumatic brain injury and status epilepticus may require deeper levels of sedation for optimal patient management [34]. Additionally, clinicians may alter the target depth of sedation based on worsening patient status that could induce iatrogenic coma [22]. It is also possible that more severely ill patients in our sample developed encephalopathy or other alterations in wakefulness regardless of sedation exposure [35]. Nevertheless, the coma-related associations we report are consistent with other reports [10–12, 36]. Interestingly, our analyses show that coma at any time during the ICU stay, as opposed to just within the first 48 h, is associated with mortality, a contrast to the results of prior investigations evaluating this relationship [10–12]. While causality cannot be inferred from these small studies, the repeated reported associations between sedative-associated coma and deleterious outcomes highlights the importance of using sedation reduction strategies like protocolization or sedation awaking trials to maintain appropriate patients at a light level of sedation [31].

Using a study cohort for our analysis that was part of a randomized, controlled trial means patients were consistently managed across the 14 study centers, delirium and coma was rigorously evaluated every 8 h by trained research personnel over the 28 days, and all study outcomes were rigorously collected [24]. The size of our study cohort is larger than most other ICU delirium-mortality studies [5–7, 10, 14–22] and allowed us to evaluate multiple covariates with the potential to affect mortality. By attempting to include only patients free of delirium at ICU admission in the analysis, our study represents a novel evaluation of whether a ‘dose-response relationship’ between incident delirium and post-ICU mortality exists, accepting that possible misclassification due to delirium symptom fluctuation may have occurred. By not being able to account for the time spent with delirium prior to ICU admission, prior studies have reported both a non-linear association [5, 14, 22] and a linear association [13, 16, 19, 21] between delirium and post-ICU mortality. Moreover, by not collecting data over a standardized period like 28 days (nor excluding patients with delirium at ICU admission) prior studies have been unable to account for the immortal time bias that patients who die before 28 days with delirium may have fewer observed days of delirium.

Our study is also strengthened by our inclusion of competing risks within the analyses, something only previously done in one other ICU delirium-mortality cohort study [6]. Compared to other delirium-mortality analyses, our study is the first to control for four well-established ICU mortality variables. Residual confounding is likely greater in these other studies given only about half have controlled for age [5–7, 10, 13, 20–22] or mechanical ventilation [5–7, 10, 13, 15, 16, 20, 21] and even fewer have corrected for sepsis [5, 6, 13, 21]. Our analysis represents the first published cohort study to consider duration of ICU stay, a factor known to influence both delirium and mortality. Our analyses also proved to be robust to the inclusion of ICU length of stay and to treatment allocation. The robustness to trial arm inclusion along with the lack of any interaction between trial arm and neurologic status further justifies the combination of all patients into a single cohort.

Several limitations need to be highlighted. The results of our analysis might not apply to patients not enrolled in the REDUCE study, although the study included patients at varying delirium risk. While patients were evaluated for delirium when maximally awake, the delirium in some patients may have been rapidly reversible thus not affecting mortality [37]. Residual confounding may exist given factors that evolve over the course of the ICU stay (e.g., severity of illness) were not considered [6]. Competing neurologic states were not considered in a time-dependent manner; future studies in this area should consider a Markov modeling approach [9]. During the period between ICU admission and study randomization (average 24 h), patients with delirium may have been coded as delirium-free if delirium fluctuated over a short period and within the eight hour period between CAM-ICU assessments. The limited data collected as part of the parent study precluded the ability to control for factors outside of routine ICU care with the potential to affect mortality such as a changing severity of illness during the ICU stay or the daily exposure to sedative infusions. Baseline severity of illness in our cohort is lower than other ICU cohorts evaluating delirium and mortality [21]. Additionally, the short study duration of mortality follow-up means a relationship between incident delirium and mortality beyond 90 days cannot be ruled out, but this appears unlikely. The coma-mortality results may be confounded by indication; patients with a greater severity of illness (and more likely to die) may require deeper sedation. However, our study controlled for baseline severity of illness and patients admitted with an acute neurologic injury (and thus more likely to develop coma) were excluded. Basing an analysis on data from a controlled, randomized trial may limit generalizability of our results to patients not included. Furthermore, we could not perform a subgroup analysis of delirium subtypes which may each have a different association with short-term mortality [38].

## Conclusion

Our study suggests that incident delirium and the days spent with it are not associated with short-term mortality within a cohort of patients who were delirium-free at ICU admission. The results of our study provide important guidance to future delirium research. Patients who develop delirium after ICU admission may have different disease trajectories than patients who already have delirium at the time of ICU admission. Competing risk factors should be considered in all delirium-mortality analysis to reduce the chance of residual confounding.

## Supplementary information

**Additional file 1.** Summary of adjusted cohort studies and RCTs evaluating the relationship between ICU delirium and mortality and how each have each have incorporated common confounders affecting this relationship. Table comparing and contrasting previous studies evaluating association between ICU delirium and mortality, with a focus on confounder adjustment.

**Additional file 2.** Sensitivity analyses exploring an interaction between neurologic status and REDUCE trial arm. This table presents the results of the sensitity analyses exploring whether an interaction exists between the REDUCE trial arm and neurologic status (delirium or coma) for each of the 8 models.

**Additional file 3.** Sensitivity analyses evaluating coma occurring in the first 48 h. Previous studies have reported an association between coma occurring in the first 48 h and mortality. This table presents the results of a similar analysis within this patient population.

**Additional file 4.** Sensitivity analyses excluding length of stay and mechanical ventilation. Additional analyses conducted to explore the impact of mechanical ventilation and ICU length of stay as mediators or confounders.

**Additional file 5.** Sensitivity analyses controlling for duration of mechanical ventilation. Additional analyses conducted to look at the effect of duration of mechanical ventilation on the associations of interest.

## Data Availability

The dataset generated and/or analyzed during the current study are not publicly but are available from the corresponding author on reasonable request.

## Abbreviations

APACHE: Acute Physiologic and Chronic Health Evaluation
CAM-ICU: Confusion Assessment Method for the ICU
CI: confidence interval
IQR: interquartile range
HR: hazard ratio
ICU: intensive care unit
RASS: Richmond Agitation Sedation Scale
SD: standard deviation
SPSS: Statistical Package for the Social Sciences
