# Supplementary material for: Association between incident delirium and 28- and 90-day mortality in critically ill adults: a secondary analysis
Source: Crit Care. 2020 Apr 20;24:161. doi: 10.1186/s13054-020-02879-6 (PMC7171767; doi:10.1186/s13054-020-02879-6)
Supplement: Supplementary file 1 — Additional file 1. Summary of adjusted cohort studies and RCTs evaluating the relationship between ICU delirium and mortality and how each have each have incorporated common confounders affecting this relationship. Table comparing and contrasting previous studies evaluating association between ICU delirium and mortality, with a focus on confounder adjustment. [file 13054_2020_2879_MOESM1_ESM.docx]

**Additional File 1.** **Summary of adjusted cohort studies and RCTs evaluating the relationship between ICU delirium and mortality and how each have each have incorporated common confounders affecting this relationship.**

| **Study** | **Baseline Delirium** | **Age** | **Severity of Illness** | **Sepsis** | **Mechanical Ventilation** | **Time-varying Covariates** |
| --- | --- | --- | --- | --- | --- | --- |
| Kishi, Gen Hosp Psych 1995 (1) | No | No | No | No | No | No |
| Dubois, ICM 2001 (2) | No | No | No | No | No | No |
| Ely, JAMA 2004 (3) | No | Yes | Baseline and daily | Yes | Yes | Yes |
| Lin, CCM 2004 (4) | No | No | Baseline | No | Yes | No |
| Thomason, CCM 2005 (5) | No | Yes | Baseline | No | Yes | Yes |
| Ouimet, ICM 2007 (6) | No | Yes | Baseline | No | No | No |
| Lat, CCM 2009 (7) | No | No | Baseline | No | Yes | No |
| Pisani, AJRCCM 2009 (8) | Yes | No | Baseline | No | No | Yes |
| Salluh, Crit Care 2010 (9) | No | No | Baseline | No | No | No |
| Shehabi, CCM 2010 (10) | Yes | Yes | Baseline | Yes | Yes | No |
| Van den Boogaard, Int J Nurs Stud 2012 (11) | Yes | Yes | Baseline | Yes | Yes | No |
| Klein Klouwenberg, BMJ 2014 (12) | Yes | Yes | Baseline and daily | Yes | Yes | Yes |
| Mehta S, CCM 2015 (13) | No | Yes | Baseline | No | Yes | Yes |
| Shehabi, CCM 2018 (14) | No | Yes | Baseline | No | Yes | No |

APACHE = Acute Physiology and Chronic Health Evaluation; ARDS = Acute respiratory distress syndrome; CAM-ICU = Confusion Assessment Method for ICU; CCI = Charlson comorbidity index; DSM-III-R = Diagnostic and Statistical Manual of Mental Disorders, Third edition, Revised; HR = Hazard ration; IADL = Instrumental Activities of Daily Living; ICDSC = Intensive Care Delirium Screening Checklist; mBDRS = modified Blessed Dementia Rating Scale; MSM = Marginal structural model; OR = odds ratio; RCT = randomized controlled trial; SOFA = Sequential Organ Failure Assessment

**References**

1. Kishi Y, Iwasaki Y, Takezawa K, et al: Delirium in critical care unit patients admitted through an emergency room. *Gen Hosp Psychiatry* 1995; 17(5):371-379

2. Dubois MJ, Bergeron N, Dumont M, et al: Delirium in an intensive care unit: A study of risk factors. *Intensive Care Med* 2001; 27(8):1297-1304

3. Ely EW, Shintani A, Truman B, et al: Delirium as a predictor of mortality in mechanically ventilated patients in the intensive care unit. *JAMA* 2004; 291(14):1753-1762

4. Lin SM, Liu CY, Wang CH, et al: The impact of delirium on the survival of mechanically ventilated patients. *Crit Care Med* 2004; 32(11):2254-2259

5. Thomason JW, Shintani A, Peterson JF, et al: Intensive care unit delirium is an independent predictor of longer hospital stay: A prospective analysis of 261 non-ventilated patients. *Critical Care* 2005; 9(4):375

6. Ouimet S, Kavanagh BP, Gottfried SB, et al: Incidence, risk factors and consequences of ICU delirium. *Intensive Care Med* 2007; 33(1):66-73

7. Lat I, McMillian W, Taylor S, et al: The impact of delirium on clinical outcomes in mechanically ventilated surgical and trauma patients. *Crit Care Med* 2009;37(6):1898-1905

8. Pisani MA, Kong SY, Kasl SV, et al: Days of delirium are associated with 1-year mortality in an older intensive care unit population. *Am J Respir Crit Care Med* 2009; 180(11):1092-1097

9. Salluh JI, Soares M, Teles JM, et al: Delirium epidemiology in critical care (DECCA): An international study. *Crit Care* 2010; 14(6):R210

10. Shehabi Y, Riker RR, Bokesch PM, et al: Delirium duration and mortality in lightly sedated, mechanically ventilated intensive care patients. *Crit Care Med* 2010; 38(12):2311-2318

11. van den Boogaard M, Schoonhoven L, van der Hoeven JG, et al: Incidence and short-term consequences of delirium in critically ill patients: A prospective observational cohort study. *Int J Nurs Stud* 2012; 49(7):775-783

12. Klein Klouwenberg PM, Zaal IJ, Spitoni C, et al: The attributable mortality of delirium in critically ill patients: prospective cohort study. *BMJ* 2014; 349:g6652

13. Mehta S, Cook D, Devlin JW, et al: Prevalence, risk factors, and outcomes of delirium in mechanically ventilated adults. *Crit Care Med* 2015; 43(3):557-566

14. Shehabi Y, Bellomo R, Kadiman S, et al: Sedation intensity in the first 48 hours of mechanical ventilation and 180-day mortality: A multinational prospective longitudinal cohort study. *Crit Care Med* 2018; 46(6):850-859
