# Supplementary material for: Association between incident delirium and 28- and 90-day mortality in critically ill adults: a secondary analysis
Source: Crit Care. 2020 Apr 20;24:161. doi: 10.1186/s13054-020-02879-6 (PMC7171767; doi:10.1186/s13054-020-02879-6)
Supplement: Supplementary file 2 — Additional file 2. Sensitivity analyses exploring an interaction between neurologic status and REDUCE trial arm. This table presents the results of the sensitity analyses exploring whether an interaction exists between the REDUCE trial arm and neurologic status (delirium or coma) for each of the 8 models. [file 13054_2020_2879_MOESM2_ESM.docx]

| **Variable** | **Mortality at 28 days** | | | | **Mortality at 90 days** | | | |
| --- | --- | --- | --- | --- | --- | --- | --- | --- |
| Model | **Incident delirium** | **Days of delirium** | **Days of coma** | **Days of delirium or coma** | **Incident delirium** | **Days of delirium** | **Days of coma** | **Days of delirium or coma** |
| Neurologic status | 0.87 (0.56-1.33) | 0.99 (0.93-1.06) | 1.17 (1.10-1.24) | 1.07 (1.03-1.12) | 0.83 (0.57-1.23) | 1.02 (0.97-1.07) | 1.09 (1.03-1.14) | 1.05 (1.01-1.09) |
| Age | 1.04 (1.03-1.06) | 1.04 (1.03-1.06) | 1.05 (1.03-1.06) | 1.04 (1.03-1.05) | 1.04 (1.03-1.05) | 1.04 (1.03-1.05) | 1.04 (1.03-1.05) | 1.04 (1.03-1.05) |
| APACHE II score | 1.07 (1.06-1.09) | 1.07 (1.06-1.09) | 1.07 (1.05-1.09) | 1.07 (1.06-1.09) | 1.07 (1.05-1.08) | 1.07 (1.05-1.08) | 1.06 (1.05-1.08) | 1.06 (1.05-1.08) |
| Sepsis present | 1.89 (1.46-2.44) | 1.87 (1.45-2.41) | 1.74 (1.34-2.26) | 1.88 (1.45-2.43) | 1.86 (1.48-2.34) | 1.85 (1.47-2.32) | 1.73 (1.37-2.19) | 1.82 (1.45-2.30) |
| Mechanical ventilation | 2.81 (1.88-4.21) | 2.77 (1.86-4.13) | 2.26 (1.51-3.39) | 2.49 (1.66-3.72) | 2.18 (1.55-3.07) | 2.14 (1.53-3.01) | 1.85 (1.31-2.61) | 1.94 (1.38-2.74) |
| ICU length of stay | 0.97 (0.95-0.99) | 0.97 (0.96-0.99) | 0.93 (0.90-0.95) | 0.95 (0.93-0.97) | 1.00 (0.99-1.01) | 1.00 (0.99-1.01) | 0.98 (0.96-0.99) | 0.98 (0.97-0.99) |
| Study Arm  Haloperidol 2mg  Haloperidol 1mg | 1.04 (0.71-1.52)  1.17 (0.74-1.85) | 1.10 (0.77-1.57)  1.21 (0.82-1.80) | 1.12 (0.79-1.58)  1.09 (0.71-1.67) | 1.25 (0.86-1.81)  1.19 (0.76-1.85) | 1.09 (0.78-1.53)  1.04 (0.68-1.60) | 1.24 (0.90-1.70)  1.26 (0.88-1.81) | 1.15 (0.84-1.58)  1.04 (0.70-1.54) | 1.28 (0.92-1.78)  1.19 (0.79-1.77) |
| Interaction with neurologic status  Haloperidol 2mg  Haloperidol 1mg | 1.13 (0.63-2.02)  1.02 (0.52-2.00) | 0.98 (0.90-1.06)  0.98 (0.89-1.07) | 1.02 (0.95-1.09)  1.04 (0.97-1.11) | 1.01 (0.96-1.06)  1.01 (0.96-1.06) | 1.21 (0.72-2.03)  1.25 (0.67-2.30) | 0.98 (0.92-1.04)  0.96 (0.89-1.04) | 1.06 (0.99-1.13)  1.04 (0.98-1.11) | 1.02 (0.97-1.06)  1.00 (0.96-1.05) |

Data is presented as hazard ratios with their associated 95% confidence intervals
