# Supplementary material for: Association between incident delirium and 28- and 90-day mortality in critically ill adults: a secondary analysis
Source: Crit Care. 2020 Apr 20;24:161. doi: 10.1186/s13054-020-02879-6 (PMC7171767; doi:10.1186/s13054-020-02879-6)
Supplement: Supplementary file 3 — Additional file 3. Sensitivity analyses evaluating coma occurring in the first 48 h. Previous studies have reported an association between coma occurring in the first 48 h and mortality. This table presents the results of a similar analysis within this patient population. [file 13054_2020_2879_MOESM3_ESM.docx]

|  | **Mortality at 28 days** | **Mortality at 90 days** |
| --- | --- | --- |
| Coma within first 48 hours | 1.12 (0.83-1.51) | 1.15 (0.87-1.53) |
| Age | 1.04 (1.03-1.06) | 1.04 (1.03-1.05) |
| APACHE II score | 1.07 (1.06-1.09) | 1.06 (1.05-1.08) |
| Sepsis present | 1.87 (1.45-2.41) | 1.84 (1.46-2.31) |
| Mechanical ventilation use | 2.56 (1.63-4.00) | 1.94 (1.32-2.86) |
| ICU Length of Stay | 0.97 (0.95-0.99) | 1.00 (0.99-1.01) |
| Study Arm  Haloperidol 2mg  Haloperidol 1mg | 1.10 (0.80-1.51)  1.18 (0.84-1.66) | 1.19 (0.90-1.57)  1.16 (0.84-1.58) |

Data is present as hazard ratios with their associated 95% confidence intervals
