# Supplementary material for: Association between incident delirium and 28- and 90-day mortality in critically ill adults: a secondary analysis
Source: Crit Care. 2020 Apr 20;24:161. doi: 10.1186/s13054-020-02879-6 (PMC7171767; doi:10.1186/s13054-020-02879-6)
Supplement: Supplementary file 4 — Additional file 4. Sensitivity analyses excluding length of stay and mechanical ventilation. Additional analyses conducted to explore the impact of mechanical ventilation and ICU length of stay as mediators or confounders. [file 13054_2020_2879_MOESM4_ESM.docx]

| **Variable** | **Mortality at 28 days** | | | | **Mortality at 90 days** | | | |
| --- | --- | --- | --- | --- | --- | --- | --- | --- |
| Model | **Incident delirium** | **Days of delirium** | **Days of coma** | **Days of delirium or coma** | **Incident delirium** | **Days of delirium** | **Days of coma** | **Days of delirium or coma** |
| Neurologic status | 0.97 (0.73-1.28) | 0.97 (0.93-1.01) | 1.08 (1.05-1.11) | 1.03 (1.01-1.06) | 1.03 (0.81-1.33) | 1.00 (0.96-1.03) | 1.08 (1.05-1.11) | 1.04 (1.02-1.06) |
| Age | 1.04 (1.03-1.06) | 1.04 (1.03-1.06) | 1.04 (1.03-1.06) | 1.04 (1.03-1.05) | 1.04 (1.03-1.05) | 1.04 (1.03-1.05) | 1.04 (1.03-1.05) | 1.04 (1.03-1.05) |
| APACHE II score | 1.07 (1.05-1.08) | 1.07 (1.05-1.08) | 1.06 (1.05-1.08) | 1.07 (1.05-1.08) | 1.06 (1.05-1.08) | 1.06 (1.05-1.08) | 1.06 (1.04-1.07) | 1.06 (1.05-1.07) |
| Sepsis present | 1.77 (1.37-2.27) | 1.77 (1.37-2.27) | 1.58 (1.22-2.04) | 1.70 (1.32-2.18) | 1.82 (1.45-2.28) | 1.82 (1.45-2.29) | 1.65 (1.31-2.08) | 1.74 (1.39-2.19) |

Data is present as hazard ratios with their associated 95% confidence intervals
