# Supplementary material for: Association between incident delirium and 28- and 90-day mortality in critically ill adults: a secondary analysis
Source: Crit Care. 2020 Apr 20;24:161. doi: 10.1186/s13054-020-02879-6 (PMC7171767; doi:10.1186/s13054-020-02879-6)
Supplement: Supplementary file 5 — Additional file 5. Sensitivity analyses controlling for duration of mechanical ventilation. Additional analyses conducted to look at the effect of duration of mechanical ventilation on the associations of interest. [file 13054_2020_2879_MOESM5_ESM.docx]

| **Variable** | **Mortality at 28 days** | | | | **Mortality at 90 days** | | | |
| --- | --- | --- | --- | --- | --- | --- | --- | --- |
| Model | **Incident delirium** | **Days of delirium** | **Days of coma** | **Days of delirium or coma** | **Incident delirium** | **Days of delirium** | **Days of coma** | **Days of delirium or coma** |
| Neurologic status | 0.92 (0.68-1.23) | 0.97 (0.92-1.01) | 1.18 (1.12-1.24) | 1.06 (1.02-1.10) | 0.96 (0.74-1.24) | 0.99 (0.95-1.02) | 1.13 (1.08-1.18) | 1.05 (1.01-1.08) |
| Age | 1.04 (1.03-1.06) | 1.04 (1.03-1.06) | 1.05 (1.03-1.06) | 1.04 (1.03-1.05) | 1.04 (1.03-1.05) | 1.04 (1.03-1.05) | 1.04 (1.03-1.05) | 1.04 (1.03-1.05) |
| APACHE II score | 1.07 (1.05-1.08) | 1.07 (1.05-1.08) | 1.07 (1.05-1.08) | 1.07 (1.06-1.08) | 1.06 (1.05-1.08) | 1.06 (1.05-1.07) | 1.06 (1.05-1.07) | 1.06 (1.05-1.08) |
| Sepsis present | 1.78 (1.38-2.30) | 1.77 (1.36-2.28) | 1.68 (1.29-2.17) | 1.77 (1.37-2.29) | 1.75 (1.38-2.20) | 1.74 (1.38-2.19) | 1.68 (1.33-2.13) | 1.74 (1.38-2.20) |
| Days of mechanical ventilation | 1.10 (1.06-1.14) | 1.10 (1.07-1.14) | 1.03 (0.98-1.07) | 1.07 (1.03-1.12) | 1.05 (1.02-1.09) | 1.05 (1.02-1.87) | 1.00 (0.97-1.04) | 1.03 (1.00-1.07) |
| ICU Length of Stay | 0.93 (0.90-0.95) | 0.93 (0.90-0.95) | 0.91 (0.88-0.94) | 0.92 (0.89-0.95) | 0.98 (0.95-0.998) | 0.98 (0.95-0.998) | 0.97 (0.95-0.99) | 0.97 (0.95-0.99) |

Data is present as hazard ratios with their associated 95% confidence intervals
